# Histone acetylations mark origins of polycistronic transcription in *Leishmania major*

**DOI:** 10.1186/1471-2164-10-152

**Published:** 2009-04-08

**Authors:** Sean Thomas, Amanda Green, Nancy R Sturm, David A Campbell, Peter J Myler

**Affiliations:** 1 Department of Genome Sciences, University of Washington, Seattle, WA 98195, USA; 2 Seattle Biomedical Research Institute, 307 Westlake Ave N. Ste 500, Seattle, WA 98109-5219, USA; 3 Department of Microbiology, Immunology, and Molecular Genetics, University of California Los Angeles, 609 E. Charles E Young Dr, Los Angeles, CA, 90095, USA; 4 Department of Global Health, University of Washington, Seattle, WA 98195, USA; 5 Department of Medical Education and Biomedical Informatics, University of Washington, Seattle, WA 98195, USA

## Abstract

**Background:**

Many components of the RNA polymerase II transcription machinery have been identified in kinetoplastid protozoa, but they diverge substantially from other eukaryotes. Furthermore, protein-coding genes in these organisms lack individual transcriptional regulation, since they are transcribed as long polycistronic units. The transcription initiation sites are assumed to lie within the 'divergent strand-switch' regions at the junction between opposing polycistronic gene clusters. However, the mechanism by which Kinetoplastidae initiate transcription is unclear, and promoter sequences are undefined.

**Results:**

The chromosomal location of TATA-binding protein (TBP or TRF4), Small Nuclear Activating Protein complex (SNAP_50_), and H3 histones were assessed in *Leishmania major *using microarrays hybridized with DNA obtained through chromatin immunoprecipitation (ChIP-chip). The TBP and SNAP_50 _binding patterns were almost identical and high intensity peaks were associated with tRNAs and snRNAs. Only 184 peaks of acetylated H3 histone were found in the entire genome, with substantially higher intensity in rapidly-dividing cells than stationary-phase. The majority of the acetylated H3 peaks were found at divergent strand-switch regions, but some occurred at chromosome ends and within polycistronic gene clusters. Almost all these peaks were associated with lower intensity peaks of TBP/SNAP_50 _binding a few kilobases upstream, evidence that they represent transcription initiation sites.

**Conclusion:**

The first genome-wide maps of DNA-binding protein occupancy in a kinetoplastid organism suggest that H3 histones at the origins of polycistronic transcription of protein-coding genes are acetylated. Global regulation of transcription initiation may be achieved by modifying the acetylation state of these origins.

## Background

### Kinetoplastid disease

Kinetoplastids are early-branching protists with unusual mechanisms of gene expression. While some are harmless free-living organisms, other members of this group infect a range of plants and animals, causing significant human disease in the form of African Sleeping Sickness (*Trypanosoma brucei*), Chagas disease (*Trypanosoma cruzi*), and leishmaniasis (*Leishmania major*), which kill approximately 400,000 people per year. The parasites are transmitted to their preferred hosts by different insect vectors where they reside and replicate as host-adapted and vector-adapted forms, respectively, with remarkably different morphologies.

*Leishmania *are transmitted by the bite of a sand fly, where they dwell in the mid-gut as promastigotes. The parasites make their way to the salivary glands where they undergo metacyclogenesis to a form infective to humans when the sand fly feeds on the victim's blood. Once inside the host bloodstream they are ingested by macrophages, where the parasites can escape the host immune system and transform into the amastigote form. Leishmaniasis symptoms depend greatly on the infecting species of *Leishmania *and present as one of three main types: a self-resolving cutaneous form, a mucocutaneous form that destroys soft tissue and cartilage in the face, and a more lethal visceral form that infects the internal organs.

### Regulation of gene expression in kinetoplastids

Kinetoplastids display peculiar molecular mechanisms, especially when it comes to gene expression. In the nucleus, functionally unrelated genes are transcribed polycistronically [1] and are processed into individual mature transcripts by *trans*-splicing, acquiring a 39-nt mini-exon from the spliced leader (SL) RNA that is attached to the 5' end of each individual messaged before it can be translated. While bacteria use polycistronic transcription as a method of co-regulating genes within an operon, kinetoplastid genes are not typically arranged by function [2], and it is thought that the steady-state levels of proteins in kinetoplastid cells are determined post-transcriptionally. The organization of genes on kinetoplastid chromosomes also reflects this high degree of polycistronic transcription; such that protein-coding genes on chromosome 1 of *L. major *are arranged in only two long gene clusters units, on opposite strands separated by a 'divergent strand-switch' region [3]. RNA polymerase II-mediated polycistronic transcription has been shown to initiate within this strand-switch region, which can also enhance expression of a marker gene two- to ten-fold [4]. Transcription also initiates within the divergent strand-switch region on chromosome 3 [5], even though it shares no obvious sequence similarity with that one chromosome 1. Little else is known about the mechanism(s) of transcription initiation for protein-coding genes in these organisms, but it appears that any promoter elements used in the process differ from those well-characterized in eukaryotic model organisms.

### Transcription in model eukaryotes

Of all the models of eukaryotic transcription initiation, the TATA box promoter serves as the most visible [6]. The TATA-binding protein (TBP) binds to the TATA box with a cadre of TBP-associated factors and, in association with general transcription factors TFIIA and TFIIB, this pre-initiation complex recruits RNA polymerase to the promoter, allowing the interaction of thee additional general transcription factors (TFIIE, TFIIF, TFIIH) that eventually initiate transcription at a defined distance from the TATA box position. Much of our mechanistic knowledge of protein-coding gene transcription in eukaryotes comes from this promoter type. However, genome-wide studies have put transcription into a more complete context, revealing that 76% of human promoters lack the canonical TATA box [7-9]. This overlaps with the larger number of promoters with initiator (Inr) elements (46%), as some promoters contain both an Inr and a TATA box. Nevertheless, some 46% of active sites of transcription initiation lack both Inr and TATA box elements. A subset of these sites is thought to represent unregulated transcription of housekeeping genes by an unknown mechanism. The new perspective provided by whole-genome studies makes it clear that much remains to be learned about the eukaryotic transcription of protein-coding genes, even in well-characterized model organisms.

Aside from protein-coding genes, a whole host of non-coding RNAs must be transcribed, with many more still being discovered [10]. Ribosomal RNAs are transcribed using promoters and recruitment factors that are highly organism-specific, with only the conserved use of RNA polymerase I in common. In contrast, tRNAs are transcribed using A/B box promoters that are conserved across all known eukaryotes, and some of the basic machinery involved in recruitment to these promoters are conserved, such as B-related proteins similar to human TFIIIB. The Small Nuclear Activating Protein complex (SNAP_c_), which in humans is a five-member complex, is involved in initiating the transcription of small nuclear (sn)RNAs from promoters containing a proximal sequence element (PSE) with or without a TATA box, depending on the gene being transcribed [11]. A minimal SNAP_c_, consisting of the three core proteins, retains its ability to initiate snRNA transcription [12], and these three proteins are indeed conserved across eukaryotes as diverse as *Drosophila *and kinetoplastids [13,14].

Eukaryotic DNA is wrapped around nucleosomes composed of H2A, H2B, H3, and H4 histone proteins, with an additional H1 histone contributing to larger order structures. By substituting histone variants, by modifying these histones, or by changing the layout of nucleosomes, eukaryotes can regulate access of chromatin to proteins involved in transcription initiation. There are a wide variety of modifications that can be made to histones, and some of these alterations can be used as markers for gene expression. In model organisms, acetylated histone H3 is found at the 5' ends of transcription start sites [15], and has been associated with an increased rate of transcription [16].

### Kinetoplastid transcription

Kinetoplastids do not employ canonical TATA box elements as part of their transcription initiation, and the Inr element used with the SL RNA promoter is widely divergent. Interestingly, *Trichomonas vaginalis*, a more early-diverging protist, uses Inr elements, suggesting that transcription from Inr-containing promoters may represent an ancient process lost in kinetoplastids [17]. However, since unregulated transcription (at promoters lacking Inr and TATA box elements) occurs in mammals as well as lower eukaryotes, it appears that they may also represent an ancestral state common to all eukaryotes. Thus, the transcriptionally-simple kinetoplastids could serve as ideal model organisms in which to study common mechanisms of unregulated transcription. If, on the other hand, the kinetoplastid system is unique, then understanding how these organisms use conserved transcription factors in functionally distinct ways may provide insights into how best to target gene expression with directed drug therapies.

There are few well-defined promoters in kinetoplastids, including promoters for non-coding RNA genes [18-20], and the unique RNA polymerase I-mediated promoters of the *T. brucei *variant surface glycoprotein and EP/PARP/procyclin genes [21], which form the basis for our current knowledge of transcription in these organisms [22]. Several proteins with similarity to conserved eukaryotic transcription initiation factors were identified through genome comparisons, and preliminary evidence for their role in kinetoplastid transcription has been demonstrated. Previous studies have shown that TBP and SNAP_50 _bind to snRNA and SL RNA gene promoters [23,24], although no evidence was found for SNAP_50 _binding to tRNA or snRNA promoters in *T. brucei *[25]. TBP was not found to bind to the rRNA promoter [24], and TBP knockdowns using RNA interference (RNAi) failed to affect rRNA levels [26]. In addition to TBP and SNAP_50_, the putative homologues of general transcription factors TFIIA [14], TFIIB [27,28], and TFIIH [29,30] all have roles in transcription. Conspicuously absent are readily identifiable homologues of TFIIE and TFIIF, of which the latter is thought to confer promoter specificity. The complement of trypanosomatid proteins annotated as transcriptionally relevant is low when compared with other organisms [2], with an almost complete lack of potential transcription factors other than those indicated above, consistent with the apparent lack of regulation of transcription initiation in these organisms.

Much of the work towards characterizing these proteins has come from binding studies and affinity purification strategies [24,27,31-35]. Only one study so far has attempted to study a kinetoplastid transcription factor on a large scale, using sequencing of precipitated chromatin to identify sequences that bind to TBP [26]. Although the sequences generated from this study could not be said to represent a systematic survey of TBP-binding sites, this study did hint that TBP interaction with the kinetoplastid chromatin was complicated.

In terms of chromatin structure, kinetoplastids possess a standard nucleosome core and their chromatin appears to undergo some sort of condensation in response to histone H1 [36], although structural adaptations likely prevent the formation of higher order structures common in other model eukaryotes [37]. Studies of epigenetic modifications in kinetoplastids have, among other accomplishments, identified acetyltransferases and found acetylated histones at a divergent strand-switch region in *Trypanosoma cruzi *[38,39]. Post-translation modifications of histones in kinetoplastids are coming increasingly into focus, and the reader is referred to the review by Horn *et al*. for a brief summary [40].

### Whole-genome maps of transcription factor occupancy

Chromatin immunoprecipitation (ChIP) has emerged as a powerful tool for analysis of interplay between chromatin structure and transcription initiation. This technique utilizes co-precipitation of DNA-binding proteins and their associated DNA sequences from live cells. In a recent adaptation of this technology (ChIP-chip), the immunoprecipitated DNA can be non-specifically amplified and hybridized to microarrays containing oligonucleotide probes tiled across the genome. Since current technology allows the creation of microarrays containing several hundred thousand to millions of oligonuceotides, ChIP-chip affords high resolution genome-wide interrogation of protein-DNA interactions on a small number of microarrays. The entire *L. major *genome is 32.8 Mbp, so the Nimblegen microarrays [41] used in this study allowed us to space 50-nucleotide probes every 85 bp across the entire genome. Using chromatin that is sheared to ~300 bp, the binding sites of a sequence-specific transcription factor can be resolved down to around ± 25 bp.

## Results

### Validation of antisera effective for *L. major *ChIP analyses

**Figure 1:**
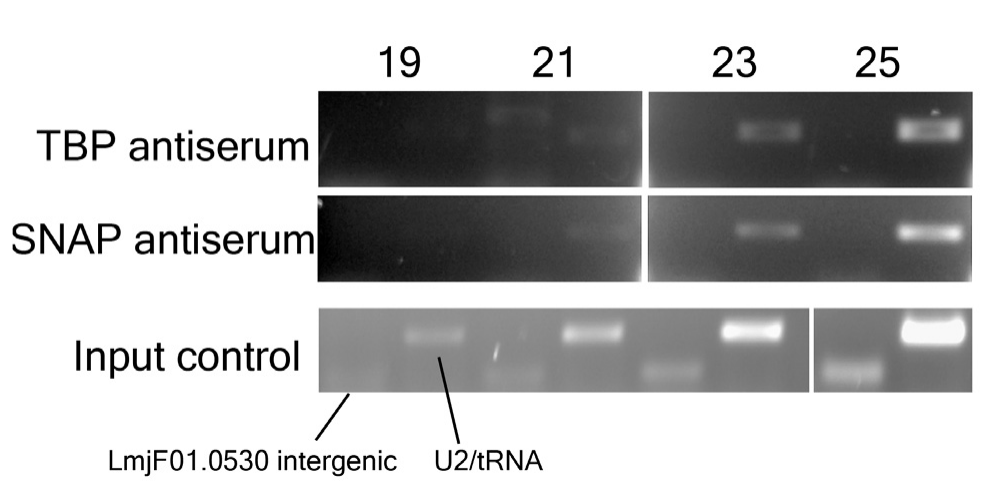
**Antisera against TBP and SNAP_50 _preferentially precipitate tRNA/snRNA sequences**. *L. major *chromatin was immunoprecipitated using antisera against TBP (top panel) or SNAP_50 _(middle panel) and PCR amplification carried out using primers specific for the U2/tRNA_Ala _locus on chr31, which is bound by both TBP and SNAP_50 _in *L. tarentolae *[24] and the *LmjF01.0530 *gene on chr1 as a control. The numbers at top represent PCR cycles. The input control in the bottom panel represents amplification of chromatin without immunoprecipitation.

Antisera raised against *Leishmania tarentolae *TBP and *L. major *SNAP_50 _have been used for ChIP of *L. tarentolae *chromatin [24], and commercial antibodies against unmodified and K_9_/K_14 _acetylated *Tetrahymena *histone H3 have been used for similar analyses in *T. cruzi *[38]. Western blot analysis using these antisera indicated that they recognized the *L. major *homologues of these targets (data not shown), although the exact residues of *L. major *H3 that are acetylated are not known. PCR assays of *L. major *chromatin precipitated using the antisera against TBP and SNAP_50 _showed enriched amplification of the U2 snRNA/tRNA promoter region when compared to a control region from within the larger chromosome (chr)1 polycistronic gene cluster (Fig 1), validating these antisera for ChIP analysis of *L. major*.

### Whole-genome occupancy maps for TBP, SNAP_50_, histone H3, and acetylated histone H3

**Figure 2:**
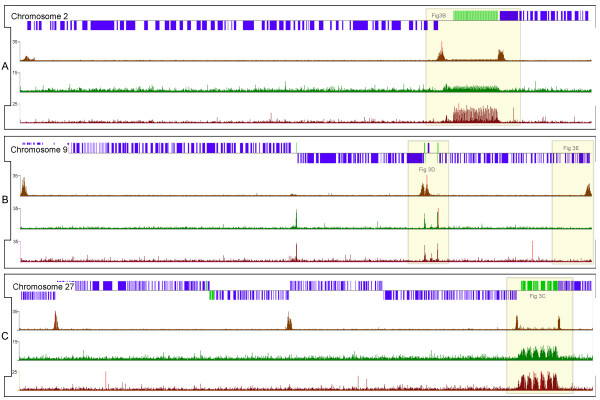
**Graphical representation of ChIP-chip results for chromosomes 2, 9 and 27**. The x-axis of each graph represents the position along the chromosome according to the genome reference sequence, and the y-axis indicates the ChIP enrichment relative to the controls. The scale in each panel is different, since the chromosomes are 357, 573 and 1130 kb in length, respectively. In each panel, the top row shows the location of protein-coding (blue), and non-coding RNA (light green) genes; while the other rows represents the ratio of acetylated:total histone H3 (brown), TBP:mock (green), and SNAP_50_:mock (red). The yellow highlights indicate regions shown in more detail in Figure 3.

*L. major *cells were cross-linked, lysed and the chromatin sheared before antisera against TBP, SNAP_50_, (histone) H3, or acetyl-H3 were used to precipitate the chromatin. The precipitated DNA was subsequently unlinked and amplified before fluorescent labeling and two-color microarray hybridization. Generally each microarray was hybridized with an experimental sample and input chromatin to control for genomic DNA copy number and biases introduced due to cross-linking, amplification, and other technical manipulations; although sometimes experimental samples were compared directly on the same array. The signal obtained for acetyl-H3 was normalized to total H3 signal and the resulting ratio at each oligonucleotide position was visualized relative to the annotated *L. major *Friedlin sequence assembly v5.0 using Nimblegen's SIGNALMAP software (see Additional file 1). The results obtained for chromosomes 2, 9, and 27 (Fig 2) reflect the overall distribution of signal intensity for the entire genome and illustrate several interesting features. The acetyl-H3:H3 ratio (shown by the brown trace on the top row under the gene map in each panel), represents the abundance of acetylated histone H3 relative to overall nucleosome levels. The ratios for TBP (green, middle row) and SNAP_50 _(red, bottom row) represent the enrichment of chromatin at each position of the chromosome when specifically precipitated with these antibodies compared to a mock precipitation. Peaks in the ChIP-chip ratios were identified using the MPEAK algorithm [42]. MPEAK relies on the fact that multiple probes will bind the same chromatin fragment if the probes are designed to be close together along the genomic sequence, with maximal binding seen where the probe sequence and the binding element overlap. The program first identifies triangular shapes with heights above a specific threshold, and then determines the exact peak position by analyzing the triangular shape. Using parameters in line with the size of sheared chromatin (~300 bp) and the distance between tiled probes used for this study (85 bp), binding peaks were predicted genome-wide for acetyl-H3, TBP and SNAP_50_. A genome-wide summary of all the acetyl-H3:H3 peaks and selected TBP/SNAP_50 _peaks can be found in Additional file 2. The following sections summarize the placements of peaks relative to important features of the genome.

### Histone H3 ChIP signal peaks are found at all divergent strand-switch regions, as well as some telomeres and a few other sites

**Figure 3:**
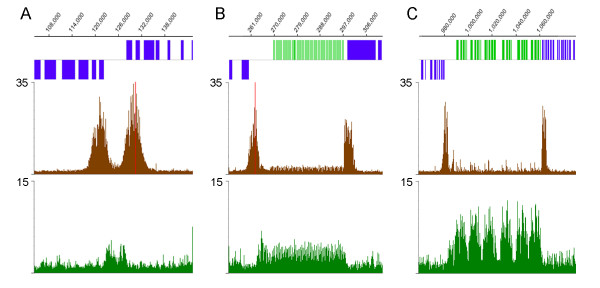
**Acetylated H3 and TBP peaks are associated with divergent strand-switch regions**. **A**. divergent strand-switch region on chr6; **B**. SL RNA locus on chr2; **C**. rRNA locus on chr27. The graphs are the same as for Fig 2, except that the SNAP_50 _results are not shown. The coordinates on each chromosome are indicated by the scale at top of each panel.

**Figure 4:**
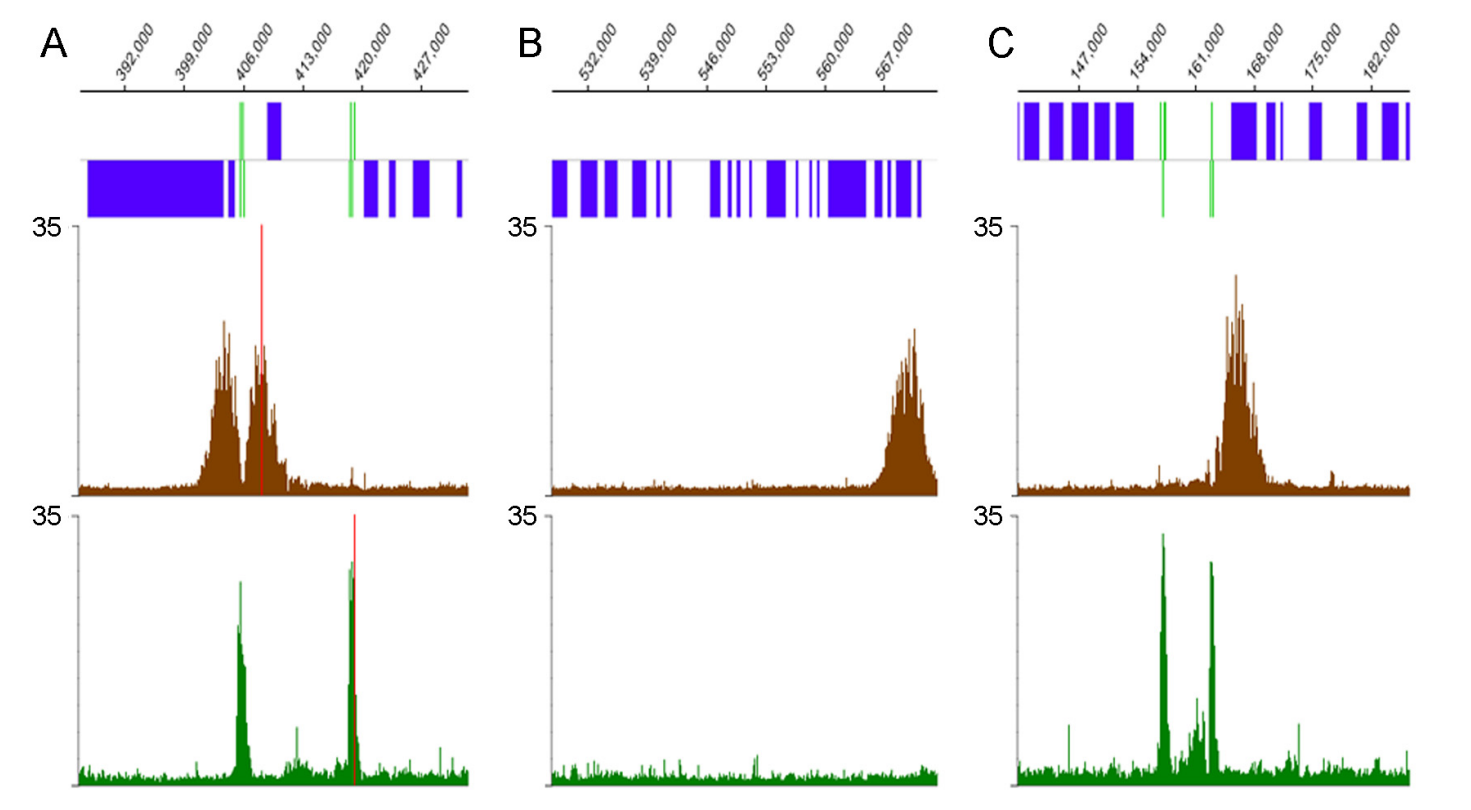
**Acetylated H3 and TBP peaks are associated with some tRNA genes and telomeres**. **A**. tRNA locus on chr9; **B**. left telomere of chr9; **C**. first tRNA locus on chr11. The graphs are the same as Fig 3.

**Figure 5:**
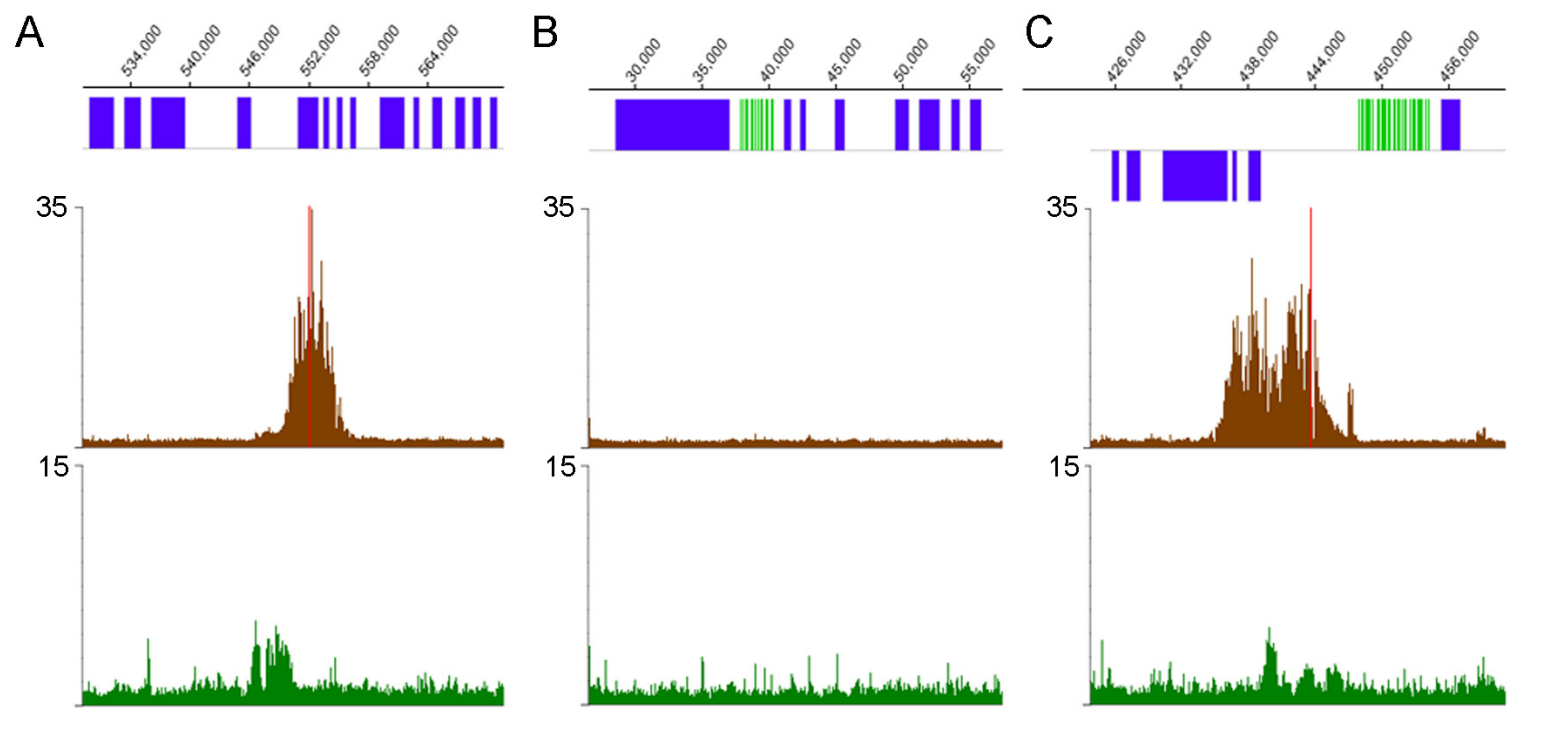
**Acetylated H3 and TBP peaks are sometimes found within polycistronic gene clusters**. **A**. putative internal transcription initiation site on chr35; **B**. snoRNA locus on chr23; and **C**. snoRNA locus on chr5. The graphs are the same as Fig 3.

Only a small number (2–13) of peaks of H3 acetylation were found on each chromosome. Only 184 peaks of H3 acetylation were found in the entire genome (not including four peaks seen within repetitive regions that probably represent normalization artifacts). Each of the 58 divergent strand-switch regions in the *L. major *genome contained peaks of H3 acetylation, as illustrated by two sites on chr27 (Fig 2) and one site from chr6 (see Fig 3A). Closer examination of the divergent strand-switch regions revealed that all but two contained two acetyl-H3 peaks. The two exceptions occurred at the 5' end of the SL RNA and rRNA arrays (see Fig 2, chr2 and chr27, respectively). In both these cases, another acetyl-H3 peak was found at the 3' end of the RNA locus at the beginning of the next polycistronic gene cluster (see Fig 3B and 3C). The only other case of RNA genes within a divergent strand-switch region is on chr9, where a tRNA cluster is flanked by two acetyl-H3 peaks (see Fig 2 and 4A). An additional 16 acetyl-H3 peaks were found at the end of chromosomes that contain a polycistronic gene cluster transcribed away from the telomere, as illustrated by the ends of chr9 in Fig 2 (and Fig 4B). Thus, every protein-coding gene cluster contains a peak of H3 acetylation at its 5' end, suggesting that these peaks correlate with transcription initiation at these sites. However, another 54 acetyl-H3 peaks were found internally within polycistronic gene clusters. These internal peaks fell into two classes: 16 were found immediately downstream of tRNA or snRNA genes (see Fig 4C for an example from chr11), but 38 were not associated with any obvious features (see Fig 5A for an example from chr17).

Each acetyl-H3 peak usually covered 4–6 kb and generally overlapped the first one or two genes in each polycistronic gene cluster, as well as the upstream intergenic region. Peaks of H3 acetylation were not observed near tRNA/snRNA clusters found within convergent strand switch regions (see chr9 in Fig 2 and Fig 4A for an example). Likewise, snoRNA clusters were not associated with acetyl-H3 peaks (see Fig 5B for an example from chr22), except in the single case on chr5 where the snoRNAs occur at a divergent strand-switch region (see Fig 5C).

### TBP and SNAP_50 _bind upstream of the peaks of H3 acetylation

**Figure 6:**
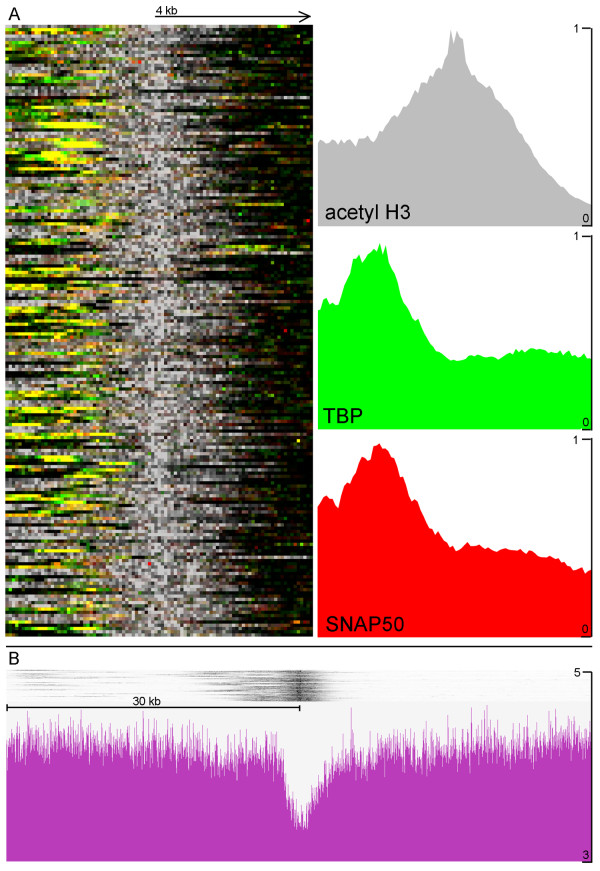
**Genome-scale representation of TBP and SNAP_50 _binding relative to H3 acetylation**. **A**. Each cell of the heatmap in the left panel represents a probe from the microarray, with each row of cells representing an 8 kb region of the genome centered on a peak of H3 acetylation aligned according to direction of transcription (indicated by the black arrow at top). The color of each cell is determined by the intensity of TBP-binding (green), SNAP_50_-binding (red), and histone H3 acetylation (grey). The top three panels to the right indicate the aggregate signal for each heatmap column for acetyl-H3 (top), TBP (middle) and SNAP_50 _(bottom). **B**. The top panel shows (black) acetylation peaks, and the bottom panel shows the average (over all regions examined) maximum G or C tract length in a 20 bp window around each position.

The great majority (174/184) of the acetyl-H3 peaks contained clear TBP-and SNAP_50_-binding sites immediately upstream (see Additional file 2). In strand-switch regions where two acetylated peaks are observed, two TBP (and SNAP_50_) peaks were usually observed (see Fig 3A), while telomeric and intergenic H3 acetylation sites were typically associated with a single peak of TBP and SNAP_50 _upstream of the acetylated region (Fig 3B and 3C). It should be noted that the TBP and SNAP_50 _peaks were generally of relatively modest intensity, except those at tRNA/snRNA clusters (Fig 4A), where they are associated with RNA polymerase III promoters, as discussed below. The TBP and SNAP_50 _signal peaks were highly correlated (0.714 Pearson's product moment correlation), indicating very similar DNA-binding patterns genome-wide, and so only the TBP peaks are shown in Figs 3, 4, 5. The peaks at tRNA/snRNA clusters were observed universally in four separate replicates performed using different starting material and performed at different times. However, the positions of other sites varied somewhat in intensity, breadth, and position between replicates. The source of this variability, either methodological or biological, is unknown. Fig 6A shows a heat-map plot of TBP and SNAP_50 _binding at all acetyl-H3 peaks within the genome. There is an apparent polarity of binding signal relative to the direction of transcription, with TBP and SNAP_50 _peaks overlapping ~1–2 kb upstream of the peak of H3 acetylation.

### H3 acetylation levels are higher in rapidly dividing cells

**Figure 7:**
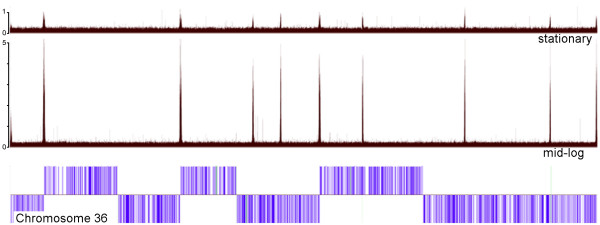
**H3 acetylation is more pronounced in rapidly-dividing than stationary phase cells**. The upper two tracks represent the ratio of acetylated histone H3 to total H3 signal from confluent stationary (day 9) cells and rapidly-dividing (day 3) cells, respectively. The vertical scale used normalizes the peak heights to those observed in stationary cells. The bottom track shows the position and strand of genes from chr36, which is 2682 kb in length.

The experiments above where performed using rapidly growing 'mid-log' procyclic promastigotes. Although kinetoplastids do not regulate transcription on the level of individual genes, it has been proposed that overall transcription rates may slow when cells approach stationary phase and begin to undergo metacyclogenesis [43]. To determine whether there were differences in the genome-wide H3 acetylation pattern associated with this phenomenon, a ChIP-chip experiment was performed using cultures that had been in stationary phase for three days (a total of 9 days after sub-culture). On average the ratios of acetyl-H3:total H3 were 5-fold higher in rapidly-growing cells than in stationary cells, even when normalized to overall histone content to control for differences between samples (see Fig 7).

### TBP and SNAP_50 _bind to the SL RNA, tRNA, snRNA, and 5S rRNA gene promoters

In contrast to the relatively binary nature of the acetyl-H3:H3 ratio (*i.e*. either an intense peak or background signal), the TBP (green, middle row of Fig 2) and SNAP_50 _(red, bottom row) ratios appear to reflect at least two different levels of binding, such that very large peaks occur at certain sites, while a number of much smaller peaks are distributed throughout the rest of the genome (often, but not always, associated with acetyl-H3 peaks).

**Figure 8:**
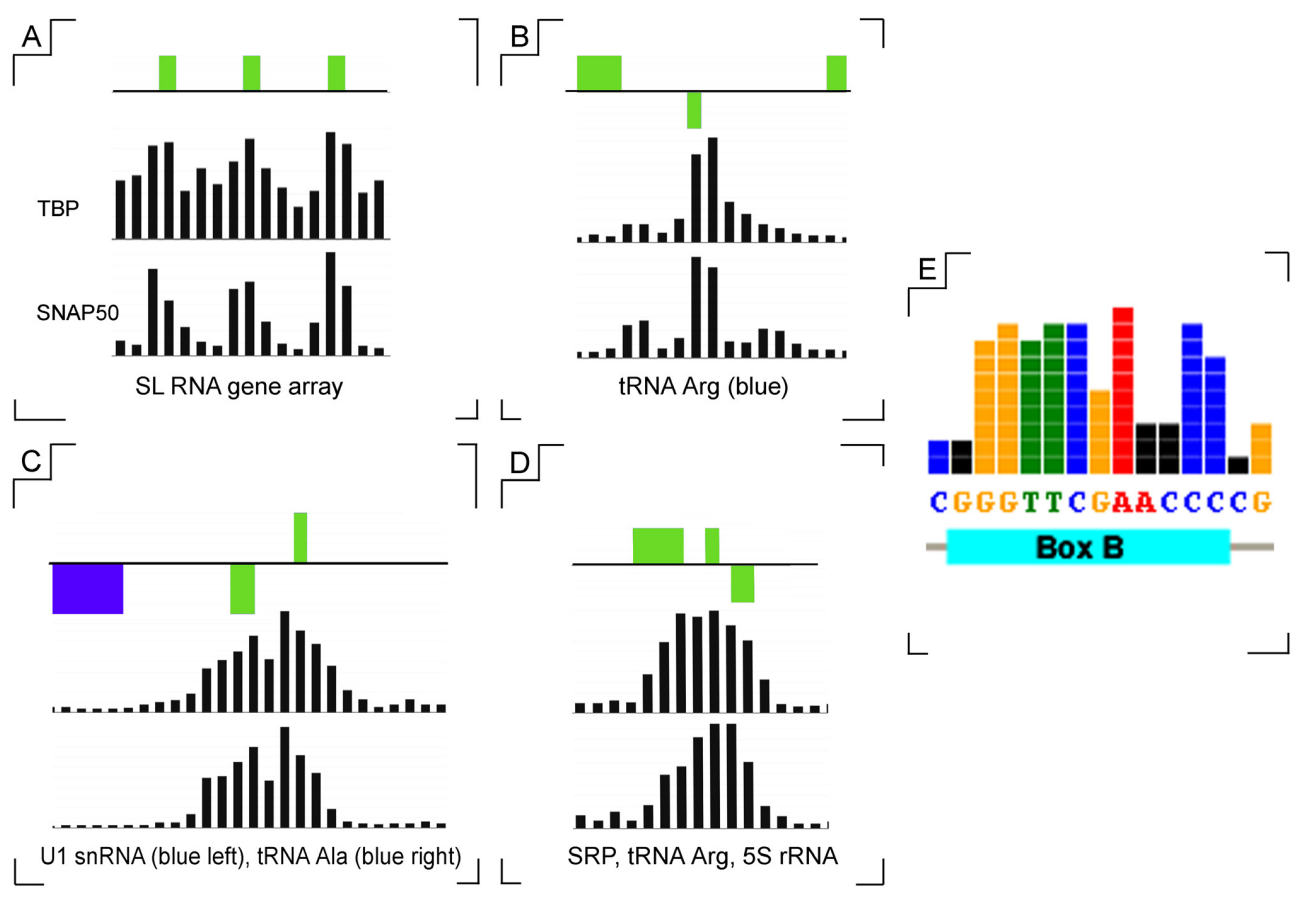
**TBP and SNAP_50 _binding to non-coding RNA promoters**. High resolution views show the TBP- and SNAP_50_-binding signal at four non-coding RNA gene loci: **A**. SL RNA locus on chr2; **B**. tRNA_Arg _locus on chr7; **C**. snRNA/tRNA locus on chr23; and **D**. SRP RNA/tRNA/5S rRNA locus on chr5. The top track in each panel shows the gene organization with protein-coding genes in blue and RNA genes in green. The middle and lower tracks represents TBP and SNAP_50 _signal, respectively, with the height of each bar representing the ratio of ChIP enrichment of specifically-precipitated chromatin to mock controls. Each black bar represents a unique probe from the microarray with a width of 50 bp, spaced at 85-bp intervals. Panel **E **shows the B Box motif recovered by MEME/MAST analysis of tRNA promoter regions.

One region associated with substantial TBP and SNAP_50_-binding peaks is the SL RNA gene locus on chr2 (see Fig 2). It should be noted that the current genome assembly puts the number of SL RNA gene copies at 120, but it is difficult to assemble nearly-identical reads into scaffolds representing correct genome sequence, especially since each SL RNA gene repeat is ~450 bp. Thus, the actual sequence of the SL RNA gene array will probably turn out to be somewhat different than what is currently included in the array design used here. Transcription factor occupancy at repetitive sequences can only be resolved as the average of binding to all genomic locations with that sequence, much in the same way that a PCR assay of ChIPed chromatin can not tell which copies of the SL RNA array the protein was binding to in order to produce a positive signal. Nevertheless, when averaged to account for the caveats above, there is a peak of TBP and SNAP_50 _binding at position -50 relative to the SL RNA gene transcription start site, centered exactly at the promoter sequence (Fig 8A).

Substantial (at least 10-fold over background) TBP and SNAP_50 _peaks were also seen 5' to all the tRNA, snRNA, and 5S rRNA gene clusters (see Fig 8B, 8C &8D for examples), consistent with binding of these transcription factors to RNA polymerase III promoters in these regions. No peaks at the 5' ends of snoRNA clusters (except on the one case on chr5, described above), consistent with the hypothesis that these RNAs do not contain individual promoters and are transcribed polycistronically with the neighboring protein-coding genes.

### Unexpected patterns of TBP and SNAP_50 _binding within rRNA genes

The most unexpected result of the ChIP-chip experiments was the pattern of TBP and SNAP_50 _binding at rRNA genes. No effect on rRNA expression was detected when TBP was knocked down due to RNA interference in *T. brucei *[26], and TBP and SNAP_50 _did not bind above background to the rRNA promoter region [44]. The data presented here clearly show TBP and SNAP_50 _binding to the rRNA locus (see chr27 in Fig 2). Surprisingly, the binding peaks correspond to rRNA coding regions and not the promoter sequences (Fig 3C). This pattern was observed in several separate precipitations and microarray hybridizations.

### Sequence motifs associated with TBP and SNAP_50_-binding

MEME and MAST software were used to identify motifs that were overrepresented in a statistically significant way at sites of TBP and SNAP_50_-binding. Three hundred nucleotides of sequence surrounding each TBP/SNAP_50 _peak was extracted and sorted into four groups representing presumed transcription initiation sites for SL RNA, rRNA, tRNA/snRNA/5SRNA, and protein-coding genes, respectively. The B-box promoter element for tRNA genes (Fig 8E) was recovered from the tRNA/snRNA/5SRNA group, indicating the validity of this approach in identifying conserved sequence motifs involved in transcription factor binding.

A single motif was identified from the presumed initiation sites for protein-coding gene transcription: a long G-tract (or C-tract in the complement). Two such G-tracts were found within the 73-bp sequence from the chr1 strand-switch region [3,4] and they are conserved across a range of *Leishmania *species [45]. While G-tracts were associated with TBP/SNAP_50_-binding sites upstream of most acetyl-H3 peaks, G-tracts of similar or longer (≥10 nucleotides) length are peppered throughout the genome, and almost half of the predicted sites of transcription initiation lack such G-tracts (data not shown). Furthermore, the positions of acetyl-H3 peaks contain shorter G-tract lengths on average than the surrounding regions (see Fig 6B). Thus, the significance of this motif in terms of TBP/SNAP_50 _binding remains an open question.

### Summary

Genome-wide ChIP-chip analysis of *L. major *promastigotes showed acetylated histone H3 peaks at the 5' ends of all polycistronic protein-coding gene clusters. These peaks occurred within all divergent strand-switch regions, at some telomeres, and at a few other sites within the gene clusters. The level of H3 acetylation was higher at these sites in rapidly growing cells than in stationary ones. Substantial TBP and SNAP_50_-binding peaks were associated with tRNA, snRNA, and SL RNA gene promoter regions, confirming previous small-scale studies. Less intense peaks were found immediately upstream of the H3 acetylation regions associated with the putative transcription initiation sites for the protein-coding genes, as has been observed in other eukaryotes. G-tracts were potentially associated with these TBP and SNAP_50_-binding regions, although similar sequences were also found in other regions of the genome.

## Discussion

More than half of all mammalian transcripts are produced from promoters with no known promoter elements – a crucial fact that went unappreciated until genome-wide studies were undertaken. Since kinetoplastid protozoa share conserved transcription factors with other eukaryotes, but lack complex transcriptional regulation, they may serve as a model for studying these mechanisms involved in non-conventional transcription initiation. On the other hand, if aspects of kinetoplastid transcription turn out to be distinct among eukaryotes, then these mechanisms could provide good targets for clinical therapy. The first whole-genome studies aimed at understanding the mechanisms of kinetoplastid transcription initiation are presented here.

The roles of TBP and SNAP_50 _in kinetoplastid transcription have been the subject of a several studies, with apparently contradictory observations. This study confirms the expected role of TBP and SNAP_50 _in binding to the SL RNA promoter, as observed in several different kinetoplastids using a variety of different experimental techniques [14,24,35,46]. There is also strong evidence that TBP and SNAP_50 _are involved in transcription of snRNAs [23,24]. However, in *T. brucei*, SNAP_50 _did not bind to an snRNA promoter under conditions where it efficiently bound the SL RNA gene promoter [25]. In the data presented here, TBP and SNAP_50 _bind universally to all snRNA promoter regions, and both proteins are found at all tRNA and 5S rRNA promoter regions as well. This contrasts with ChIP data from *L. tarentolae*, where only TBP was observed binding to those sites [24]. Without similar whole-genome studies in these other kinetoplastid model organisms, the apparent contradiction in all of the available data is difficult to resolve.

With regards to rRNA transcription, TBP knockdown by RNA interference in *T. brucei *had no effect on steady-state rRNA levels [26], and TBP or SNAP_50 _did not bind above background to the rRNA promoter region [24], even though an element of the *T. brucei *rRNA promoter is reported to bind SNAP_50 _*in vitro *[47]. The data here show both TBP and SNAP_50 _apparently bind within the rRNA gene coding sequence, but not the promoter. One possible explanation is that it may represent precipitation of rRNAs along with TBP/SNAP_50 _nascent polypeptides, although how this RNA would be labeled and why it was not also seen with H3 is not clear. Alternatively, the apparent pattern of binding may be merely an artifact caused by repetitive sequences, although this does not appear to be the case for the SL RNA locus. However, transcription of rRNAs is notably distinct from organism to organism even among closely-related crown-group eukaryotes, and species-specific effects may explain the apparent differences between *T. brucei *and *L. major*. With key components still being identified [31], the full story of RNA polymerase I transcription in kinetoplastids is clearly yet to be written.

K_9_/K_14 _acetylation of histone H3 is a marker for sites of active transcription initiation in other eukaryotic systems. Our observation that similar H3 acetylation is found at all divergent strand-switch regions, as well as a few other sites throughout the *L. major *genome, in a polarity consistent with expected direction of transcription, suggests that this acetylation represents the first marker for sites of RNA polymerase II-mediated transcription initiation of protein-coding genes in kinetoplastids. This conclusion is bolstered by our finding that peaks of TBP binding were observed immediately upstream of the vast majority of acetylated regions. This suggests that histone acetylation is a marker for open chromatin, which makes specific regions of the genome available for binding of transcription initiation complexes, as observed in other organisms. Since TBP and SNAP_50 _signals were correlated genome-wide, and both were associated with H3 acetylation at the 5' end of polycistronic gene clusters, it seems likely that SNAP_50 _has a role as a general transcription factor in kinetoplastid transcription, an adaptation that would be unique among all model eukaryotes.

The hypothesis that kinetoplastids regulate overall transcription rates according to cell density [43] led to the idea that the mechanism could involve changes in chromatin structure. If histone acetylation serves as markers for transcriptional availability, then one should observe a decrease in acetylation levels in stationary cells when compared to rapidly-dividing ones. Our observation that acetyl-H3 peaks are considerably reduced in magnitude in stationary stage cells supports the hypotheses that kinetoplastids do regulate overall transcription rates.

Aside from identifying the sites of transcription initiation, one of the main goals of this research is to identify DNA elements responsible for recruiting RNA polymerases to those sites. Several different methods have been used successfully in other organisms to identify regulatory elements from an enriched selection of sequences likely to contain similar elements. MEME and MAST analysis of sequences at sites of enhanced ChIP signal easily recovered known tRNA promoter elements; but no clear motifs were discovered for protein-coding gene transcription initiation sites. The only identifiable motif found in a majority of the isolated sequences was a G-tract (or C-tract) longer than 10 nucleotides. Similar elements were found within the transcription-enhancing 73-bp sequence derived from the *L. major *chr1 strand-switch region [4] which is conserved across *Leishmania *species [45]. However, because of their ubiquitous presence in regions devoid of TBP and acetylated H3 peaks, it is unlikely that these elements are sufficient to direct transcription initiation. Furthermore, the breadth of the TBP and SNAP_50 _peaks is not indicative of typical promoter-directed initiation from a single initiation point. This is consistent with the finding that the chr1 strand-switch region contains several distinct transcription sites in both directions [4].

Previous bioinformatic analyses of several divergent strand-switch regions from *L. major *revealed an unusually high AT composition, a lack of putative hairpins and a strong curvature of the DNA [48]. Our data indicate that the peaks of acetylated H3 are associated with increased AT content ~1–2 kb downstream of the G/C-tracts above. The mechanistic implications of this finding are not yet clear, although it is tempting to speculate that it may be associated with enhanced melting of the DNA strands during transcription initiation. Local bending of the divergent strand switch regions could allow access to transcription initiation [49] and binding of proteins to DNA can drastically alter the shape of the DNA in ways that can increase curvature or facilitate secondary structures. In mapping predicted curvature genome-wide, it is clear that the divergent strand-switch regions do possess greater predicted curvature based on the dinucleotide stacking models used. However, other regions not associated with ChIP signal peaks also possess predicted curvatures of similar magnitudes. Thus, while DNA secondary structures and curvature may play a role in kinetoplastid transcription, the mechanisms by which this may happen elude current bioinformatics predictions. Even with ChIP data suggesting that a protein binds to a given region, the induced bending that a protein will cause cannot be predicted without doing very involved *in vitro *bending assays or resolving the structure of the protein-bound DNA. Other secondary structures, like triple helices for example, are difficult to predict bioinformatically at present.

## Conclusion

The use of ChIP-chip analysis to probe genome-wide transcription factor occupancy has been applied for the first time to the study of kinetoplastids. The results confirm many conclusions made from previous small-scale studies and suggest that there are only 184 transcription-initiation sites for protein-coding genes in *L. major*. They also extend our understanding of the roles of TBP and SNAP_50 _in *L. major *transcription. TBP and SNAP_50 _appear to bind to all RNA polymerase II and III promoters and appear to have identical binding patterns genome-wide, laying open the interesting possibility that SNAPc may serve as a general transcription factor for protein-coding transcription in these organisms. The identification of acetylated H3 histones at divergent strand-switch regions and at a few other sites throughout the genome suggests that chromatin accessibility may restrict transcription initiation of protein-coding genes in kinetoplastids to a few select sites throughout the genome. Changes in acetylation between rapidly-dividing cells and stationary ones may constitute evidence of wholesale regulation of transcriptional rates by modulation of chromatin availability at these sites. Future whole-genome studies of epigenetic features and chromatin remodeling proteins are likely to shed more light on the mechanisms of kinetoplastid transcription initiation. The transcriptional machinery of kinetoplastids is highly reduced, making complete binding maps of all known kinetoplastid transcription factors and epigenetic factors likely within the near future once suitable antibodies are developed.

## Materials and methods

### Chromatin Immunoprecipitation (ChIP)

ChIPs were performed as previously described [24], except that *L. major *cells were used instead of *L. tarentolae*. For each IP, 2.5 × 10^9 ^*L. major *cells were incubated with 1% formaldehyde at 25°C for 15 min. Next, 2.5 ml of 2.5 M glycine was added and incubation continued at 25°C for an additional 5 min. The cells were pelleted and washed once with 20 ml 1× phosphate buffered saline (PBS), pelleted again and washed once more with 2 ml PBS. After the final pelleting, the cells were re-suspended in 400 μl ChIP lysis buffer (50 mM HEPES, pH 7.5, 140 mM NaCl, 1% Triton X-100, 0.1% sodium deoxycholate and Complete Protease Inhibitor Tablets [Roche]) and an equal volume of glass beads was added. The sample was shaken continuously for 30 min on a vortex at 4°C. The lysate was recovered by piercing the bottom of the tube and collecting drops. The samples were sheared on a Misonix Microson model #XL2007 sonicator with 6–10 s pulses at 12 Watts, followed by a 15 s pause after each pulse. The lysate was centrifuged at 10,000 rpm for 10 min at 4°C. The supernatant was taken and its protein concentration determined. One mg of protein was used for each immunoprecipitation reaction. One-fortieth of the amount of lysate that would be used for an immunoprecipitation was reverse cross-linked (65°C overnight), and the DNA was purified on a Qiagen QiaQuick^® ^spin column and analyzed by agarose gel electrophoresis to determine the average genomic fragment size achieved by sonication (300 bp).

Commercial antisera against peptides representing *Tetrahymena *unmodified (ab12079, Abcam) and H_9_/K_14_-acetylated (06–599, Upstate) histone H3 sequence were used to precipitate chromatin that was subsequently amplified and used to create fluorescently labeled probes used in two-color microarray hybridization, as were antisera against recombinant *L. tarentolae *TBP and *L. major *SNAP_50 _[24]. Each antiserum (equilibrated in lysis buffer) was added to 1 mg of lysate along with 50 μl protein A-agarose and incubated overnight at 4°C. Two washes each of the following were then performed, pelleting beads after each wash: 1 ml ChIP lysis buffer, 1 ml high salt lysis buffer (same as lysis buffer except 500 mM NaCl), 1 ml ChIP wash buffer (10 mM Tris-HCl pH 8.0, 250 mM LiCl, 0.5% NP-40, 0.5% sodium deoxycholate, 1 mM EDTA), and 1 ml TE. The beads were mixed with 75 μl of ChIP elution buffer (50 mM Tris-HCl pH 8.0, 1% SDS, 10 mM EDTA) and the sample incubated at 65°C for 10 min. After centrifugation, the supernatant was collected and the beads eluted again with 75 μl elution buffer. The supernatants were combined and incubated at 65°C overnight to reverse the cross-linking and the liberated DNA was purified with a QiaQuick column. Where PCR is used to assay the chromatin, 1 μl is used for each 10 μl reaction. Chromatin to be used in microarray experiments was lyophilized before amplification using the GenomePlex Whole Genome Amplification Kit (Sigma) to obtain 4 μg of DNA. The amplified DNAs were sent to Nimblegen for two-color labeling and hybridization using their standard ChIP-chip protocol [50]. Following hybridization, the microarrays were scanned using a GenePix 4000B Scanner and data processed according to standard Nimblegen methodology, before being returned for further analysis.

A ChIP sample and a control sample were generated for each experiment. For the TBP and SNAP_50 _experiments, 'mock' immunoprecipitations were performed in which input chromatin was subjected to immunoprecipitation conditions without the use of antiserum. For acetyl-H3 experiments, the antiserum against unmodified H3 peptides was used as a control.

### Microarray design

The 32.8 Mbp *L. major *genome assembly (version 5.0) was used to design 50-nt-long tiled probes interspersed by 35-bp gaps, yielding a final custom-designed Nimblegen microarray containing 387,865 'top strand' probes (as determined by the orientation of the v5.0 genome sequence available at the Sanger website). The probe design data and all microarray data are available from the NCBI Gene Expression Omnibus (Accession number GSE13415).

### Analysis of microarray data

The microarray data was analyzed in raw form using EDITPAD PRO or the R statistical package, and viewed graphically (from a gff file format) using Nimblegen's SIGNALMAP software. A combination of R and PERL scripts were written for microarray data analysis. Scripts for these analyses are available upon request. All experiments were performed in triplicate, using different cell cultures, except for the comparison of mid-log *versus *stationary cultures, which was done only once.

### Peak identification, motif discovery, and curvature analysis

The MPEAK algorithm was used to identify peaks in the ChIP data [42]. The program identifies significant peaks in the data and applies a model of chromatin shearing to determine how likely it is that the point truly represents a peak based on the signals of probes nearby in sequence-space. A PERL script was written to extract the 300 nucleotides surrounding each MPEAK-predicted TBP/SNAP_50 _peak and the resulting sequences were divided into different groups. Sequences representing peaks near SL RNAs, and rRNA genes were put into two different bins; those representing tRNA, 5S rRNA, and snRNA genes were put into a third bin; while a fourth bin represented all other peaks. A fifth bin represented peaks found within divergent strand-switch regions, and a final bin represented a random selection of sites throughout the genome as a control. The sequences in each bin were submitted to the MEME/MAST algorithm for motif discovery, and the resulting motifs were analyzed manually to recover already identified promoter elements and to determine whether any of the proposed motifs represented a newly discovered promoter element. For analysis of G-tract distribution, a PERL script was written to systematically scan the genome sequence and count the number of uninterrupted Gs (or Cs) beginning at each base and to report the maximum length of G or C tracts in a window, this was used to demonstrate the lower average length of maximum G and C tracts at sites of H3 acetylation. A separate PERL script was written to measure inherent curvature of the genomic sequence based on previously published algorithm using a dinucleotide stacking model [49]. This model assumes B-DNA structure and does not account for bending that might arise due to alternate conformations, complex secondary structures, or protein binding. This script is available upon request.

## Abbreviations

(ChIP): Chromatin immunoprecipitation; (chr): chromosome; (rRNA): ribosomal RNA; (SL RNA): spliced leader RNA; (SNAP_50_): small nuclear activating protein complex subunit 50; (snRNA): small nuclear RNA; (snoRNA): small nucleolar RNA; (tRNA): transfer RNA; (TBP): TATA-binding protein.

## Authors' contributions

ST participated in the study design, optimized the ChIP-chip process, performed the computational analysis of the microarray data, and drafted the manuscript. AG carried out cell culture, antibody analysis, chromatin immunoprecipitation and preparation for microarray hybridization. DC and NS provided the antisera used in the study and made substantial contributions to writing the manuscript. PM conceived of the study, directed its design and analysis, and made substantial contributions to the manuscript.

## Supplementary Material

Additional file 1 **Genome-wide distribution of acetylated histone H3, TBP, and SNAP_50_**. The ratio of ChIP enrichment for histone H3 acetylations, TBP-binding, and SNAP_50_-binding are presented relative to input control for all 36 chromosomes, in similar manner to Fig 2. Also included on the bottom row of each figure is the repeat number of each oligonucleotide probe. On the top track, protein-coding genes and non-coding RNAs are shown in blue and green, respectively. A red bar at any position indicates that the value of the signal at that position is greater than the maximum value shown on the graph. Click here for file

Additional file 2 **Predicted sites of polycistronic transcription initiation in *L. major***. Each acetylated histone H3 peak region is described in tabular form, listing the chromosome; the approximate boundaries of the region; location of the signal peak; location(s) of associated TBP/SNAP_50 _peak(s); a systematic name; type (see below); strand (T for top, B for bottom); number of genes in the associated polycistronic gene cluster; and a comment noting any other features (*e.g*. RNA genes) associated with the entry. The codes used to describe the type of peak are D (divergent strand-switch region), C (convergent strand-switch region), T (telomeric), R (downstream of an RNA gene cluster); and I (internal within a polycistronic gene cluster). The total number of each peak type is shown at the bottom of the table. Click here for file
