# Supplementary material for: Histone acetylations mark origins of polycistronic transcription in Leishmania major
Source: BMC Genomics. 2009 Apr 8;10:152. doi: 10.1186/1471-2164-10-152 (PMC2679053; doi:10.1186/1471-2164-10-152)
Supplement: Additional file 2 — Predicted sites of polycistronic transcription initiation in L. major. Each acetylated histone H3 peak region is described in tabular form, listing the chromosome; the approximate boundaries of the region; location of the signal peak; location(s) of associated TBP/SNAP50 peak(s); a systematic name; type (see below); strand (T for top, B for bottom); number of genes in the associated polycistronic gene cluster; and a comment noting any other features (e.g. RNA genes) associated with the entry. The codes used to describe the type of peak are D (divergent strand-switch region), C (convergent strand-switch region), T (telomeric), R (downstream of an RNA gene cluster); and I (internal within a polycistronic gene cluster). The total number of each peak type is shown at the bottom of the table. [file 1471-2164-10-152-S2.pdf]

chr	location	H3Ac peak(s)	TBP/SNAP	name	Type	strand	genes	Comments
1	74-78K	76049	78K	1.1L	D	B	31	
1	78-84K	79789	78K	1.1R	D	T	53	
1	266-268K	267451		1.2?				repetitive_262-269K_LST_repeats
2	3-9K	4057		2.1?				repetitive_H2/SCG_repeat
2	260-265K	262492	264-266K	2.2	D	B	58	5'_to_SL_array
2	297-302K	298848	297-298K	2.3	R	T	17	3'_to_SL_array
3	0-5K	3744	5K	3.1L	D	B	1	
3	5-10K	7168	5K	3.1R	D	T	64	
3	252-257K	254686	247-252K	3.2	I	T	1	
3	378-383K	380468 & 382084	383-384K	3.3	T	B	30	
4	0-6K	3080	1K	4.1	T	T	40	
4	261-266K	262884	266K	4.2	I	B	26	
4	467-472K	469916	473K	4.3	T	B	60	
5	1-10K	5097	4K	5.1	T	T	44	
5	146-152K	148379	146-148K	5.2	I	T	52	
5	380-385K	382134	?	5.3L	D	B	8	
5	385-390K	386554	?	5.3R	D	T	8	
5	435-440K	438354	440K	5.4L	D	B	9	
5	440-446K	443456	440K	5.4R	D	T	2	5' to snoRNAs
6	117-123K	121299	123-125K	6.1L	D	B	36	
6	127-133K	130230	125K-127K	6.1R	D	T	19	
6	195-201K	198062	?	6.2	I	T	76	
6	500-504K	502529	504K	6.3L	D	B	4	
6	504-508K	508564	504K	6.3R	D	T	1	repeats_at_right
7	5-10K	8460	10K	7.1L	D	B	1	
7	10-15K	12006	10K	7.1R	D	B	15	
7	205-210K	207365	210K	7.2L	D	B	33	
7	214-219K	215867	214K	7.2R	D	T	37	
7	382-387K	382778 & 384624	384K	7.3.1	R	T	40	3'_to_tRNA
7	590-595K	591281		7.4?				repetitive_H2/SCG_repeat
8	1-6K	2625	1K	8.1	T	T	89	
8	481-489K	485059	488-492K	8.2L	D	B	23	
8	491-497K	493764	488-492K	8.2R	D	T	23	
9	2-7K	2977 & 4847	0-2K	9.1	T	T	82	
9	400-406K	403532	406K	9.2L	D	B	31	3'_to_tRNA_cluster_at_405.5K
9	406-411K	408207	406K	9.2R	D	T	1	5'_to_tRNA_cluster_at_419K
9	567-572K	569840	572K	9.3	T	B	54	
10	14-19K	18300	19K	10.1L	D	B	3	
10	19-24K	21530	19K	10.1R	D	T	54	
10	291-297K	293819	297K	10.2	I	B	9	
10	528-534K	530993	534K	10.3L	D	B	66	5'_to_tRNA_cluster_at_534K
10	534-539K	535243	534K	10.3R	D	T	10	3'_to_tRNA_cluster_at_534K
11	1-6K	3161	0-1K	11.1	T	T	47	
11	164-169K	165722	157-163K	11.2	R	T	51	3'_to_tRNA_clusters_at_157K_and_163K
11	382-387K	384362	382K	11.3	R	T	44	3'_to_tRNA_at_382K
12	378-385K	382820	385/387K	12.1L	D	B	86	
12	390-395K	392935	390K	12.1R	D	T	12	
12	667-675K	673076	675K	12.2	T	B	41	
13	137-145K	141069	144K	13.1L	D	B	45	
13	149-154K	148915	147K	13.1R	D	T	25	
13	482-487K	484887	486K	13.2	I	B	68	
13	639-647K	643198	645K	13.3L	D	B	31	
13	643-650K	646088	645K	13.3R	D	T	2	
14	0-9K	4240	2K	14.1	T	T	48	
14	420-427K	423490	?	14.2L	D	B	61	
14	427-433K	429015	?	14.2R	D	T	46	
15	83-89K	86814	87K	15.1L	D	B	25	
15	87-94K	90129	87K	15.1R	D	T	49	snoRNAs_at_160K,_tRNA_cluster_at_323K
15	618-625K	622156	623K	15.2L	D	B	94	
15	624-629K	626151	623K	15.2R	D	T	1	
16	334-341K	337001	340-344K	16.1L	D	B	95	
16	344-349K	346143	340-344K	16.1R	D	T	24	
16	644-650K	647167	649K	16.2L	D	B	41	
16	647-653K	649571	649K	16.2R	D	T	14	
17	162-167K	165084	167K	17.1	I	B	40	
17	335-342K	338136	342K	17.2	R	B	36	5'_to_tRNA_cluster_at_345K
17	410-416K	413305	416K	17.3L	D	B	20	
17	415-422K	417130	416K	17.3R	D	T	62	
18	225-231K	228189	231K	18.1L	D	B	56	snoRNAs_at_180K
18	231-236K	231504	231K	18.1R	D	T	49	
18	449-455K	451405	449K	18.2	T	T	66	
19	65-72K	68867	?	19.1L	D	B	22	
19	65-72K	69632	?	19.1R	D	T	47	
19	304-307K	305179	?	19.2	I	T	84	
19	632-643K	635407 & 638977	637K	19.3	I	T	26	possible_genome_annotation_problem
20	94-99K	96449	99K	20.1L	D	B	26	
20	99-104K	102144	99K	20.1R	D	T	54	snoRNAs_at_320-340K
20	349-354K	350579	348K	20.2	I	T	35	
20	527-533K	528764 & 530100	526K	20.3	I	T	31	peak_split_at_529K
20	735-742K	738382	741K	20.4	T	B	30	
21	3-8K	4560	10K	21.1L	D	B	1	
21	8-15K	11955	10K	21.1R	D	T	54	tRNA_cluster_at_160K
21	214-219K	218477	220-224K	21.2L	D	B	20	
21	223-230K	225872	220-224K	21.2R	D	T	56	443K
21	720-726K	724750	726K	21.3L	D	B	77	
21	725-731K	728490	726K	21.3R	D	T	17	
22	5-11K	7610	10K	22.1	I	B	1	
22	316-324K	321526	323K	22.2L	D	B	76	
22	324-329K	324841	323K	22.2R	D	T	43	
22	602-607K	605340	607-610K	22.3L	D	B	26	snoRNAs_at_587K
22	611-616K	612413	607-610K	22.3R	D	T	21	
23	2-9K	3010	2K	23.1	T	T	60	snoRNAs_at_40K
23	215-221K	218147	215K	23.2	R	T	4	5'_to_tRNA_cluster_225-227K
23	545-551K	548719	551K	23.3L	D	B	73	
23	554-559K	555308	554K	23.3R	D	T	60	
24	0-8K	4976	3K?	24.1	T	T	63	
24	230-236K	232482	230K	24.2	I	T	57	
24	469-476K	471731	469K	24.3	I	T	35	tRNA_cluster_at_623K
24	713-718K	715556	717K	24.4	I	B	28	snoRNA_at_682K
24	830-836K	833036	836K	24.5L	D	B	70	
24	835-841K	837350	836K	24.5R	D	T	1	
25	263-269K	266266	268K	25.1L	D	B	73	
25	267-273K	269132	268K	25.1R	D	T	38	
25	582-588K	585178	588K	25.2	I	B	40	snoRNAs_at_623-632K
25	823-828K	824331	827K	25.3L	D	B	78	
25	827-833K	828666	827K	25.3R	D	T	30	
25	903-913K	907131		25.4?				repetitive
26	224-230K	228362	230K	26.1	I	B	76	
26	303-310K	306910	309K	26.2L	D	B	24	
26	308-313K	310990	309K	26.2R	D	T	55	
26	575-580K	576440	575K	26.3	I	T	11	
26	610-620	610080-620328	608K	26.4	I	T	62	repetitive_FAD_genes_at_610-620K, snoRNAs_at_648-677K
26	919-924K	920327	919K	26.5	I	T	45	
27	69-73K	71699	73K	27.1L	D	B	29	

27	73-79K	74481	73K	27.1R	D	T	63	snoRNAs_at_370K
27	526-534K	530485	534K	27.2L	D	B	37	snoRNAs_at_375-385K
27	534-540K	534225	534K	27.2R	D	T	48	
27	978-984K	981145	984K	27.3	D	B	65	5'_to_rRNA_gene_array
27	1062-1066K	1063782	1062K	27.4	R	T	25	3'_to_rRNA_gene_array
28	0-8K	3830	3K	28.1	T	T	35	
28	275-282K	279142	282K	28.2L	D	B	44	
28	281-286K	283222	282K	28.2R	D	T	85	
28	815-822K	819429	822K	28.3L	D	B	53	
28	823-829K	825897	823K	28.3R	D	T	63	
28	1153-1158K	1155774	1158K	28.4	I	B	34	
29	335-341K	338616	341K	29.1L	D	B	92	
29	344-350K	345586	344K	29.1R	D	T	57	
29	813-820K	816267	820K	29.2	R	B	41	3'_to_tRNA_cluster_at_820K
29	1025-1033K	1031210	1033K	29.3L	D	B	52	
29	1033-1037K	1035035	1033K	29.3R	D	T	43	
30	221-229K	226815	229K	30.1L	D	B	73	snoRNAs_at_145K
30	231-239K	234048	231K	30.1R	D	T	121	snoRNAs_564K
30	644-649K	644142	644K	30.2	I	T	35	
30	1090-1099K	1093737 & 1095413	?	30.3	I	B	80	peak_split_at_1094K
30	1231-1237K	1234347	1237K	30.4L	D	B	42	peaks_do_not_correspond_exactly_ to_strand_switch_region, likely_genome_annotation_problem
30	1237-1243K	1237832	1237K	30.4R	D	T	52	peaks_do_not_correspond_exactly_ to_strand_switch_region, likely_genome_annotation_problem
31	197-204K	201084	204K	31.1	R	B	62	3'_to_tRNA_cluster_at_204K
31	490-494K	492195 & 493640	494K	31.2	R	B	66	3'_to_tRNA_cluster_at_495K
31	773-779K	774370 & 777005	779K	31.3	I	B	43	
31	971-976K	974913	976K	31.4	I	B	35	
31	1308-1315K	1313683	1316K	31.5	R	B	75	3'_to_tRNA_cluster_at_1316K
31	1469-1473K	1471370	1473K	31.6L	D	B	50	
31	1473-1477K	1474600	1437K	31.6R	D	T	3	
32	180-185K	182930	185K	32.1L	D	B	50	
32	185-191K	187435	185K	32.1R	D	T	93	
32	835-841K	839005	841K	32.2	I	B	84	
32	1163-1166K	1166362	1166-1173K	32.3L	D	B	85	
32	1173-1177K	1173772	1166-1173K	32.3R	D	T	104	
33	103-107K	104718	107K	33.1	R	B	31	3'_to_tRNA_cluster_at_107K, snoRNAs_at_103K&90-93K
33	259-265K	263123	265K	33.2L	D	B	46	
33	265-271K	267288	265K	33.2R	D	T	95	snoRNAs_at_308K
33	601-609K	603759	601K	33.3	R	T	30	5'_to_tRNA_at_601K
33	765-772K	767671	763-765K	33.4	I	T	14	snoRNAs_at_676K
33	812-817K	815842	817K	33.5L	D	B	4	
33	817-824K	819412	817K	33.5R	D	T	112	
33	1336-1342K	1337571	?	33.6	I	T	39	
34	0-7K	5710	7K	34.1L	D	B	1	
34	7-13K	9450	7K	34.1R	D	T	70	
34	317-322K	321507	322K	34.2L	D	B	2	
34	322-329K	324203	322K	34.2R	D	T	39	
34	556-563K	559566	563K	34.3	I	B	20	
34	1150-1155K	1153249	1155K	34.4L	D	B	136	
34	1155-1163K	1156989	1155K	34.4R	D	T	100	
34	1535-1540K	1537421	1535K	34.5	R	T	112	3'_to_tRNA_cluster_at_1535K
35	39-47K	41505 & 45500	47K	35.1L	D	B	19	
35	47-52K	48815	47K	35.1R	D	T	66	
35	396-403K	398829	396K	35.2	I	T	32	
35	550-556K	552386	550K	35.3	I	T	27	
35	731-738K	735069	738K	35.4L	D	B	26	
35	738-741K	738615	738K	35.4R	D	T	40	
35	875-880K	876763	875K	35.5	I	T	44	
35	1399-1405K	1402117	1405K	35.6	I	B	88	snoRNAs_at_1261-1265K&1331K
35	1537-1543K	1540881	1543K	35.7L	D	B	43	
35	1543-1549K	1545192	1543K	35.7R	D	T	79	snoRNAs_at_1671-1681K
35	1853-1860K	1854996	1853K	35.8	I	T	66	
35	2067-2073K	2069321	2067K	35.9	I	T	1	
36	149-155K	153406	155K	36.1L	D	B	55	
36	155-161K	156721	155K	36.1R	D	T	83	
36	774-780K	777021	780K	36.2L	D	B	63	
36	779-785K	780676	780K	36.2R	D	T	63	snoRNAs_at_940-943K
36	1103-1110K	1110172	1110-1116K	36.3	I	B	17	snoRNAs_at_1078-1079K
36	1232-1239K	1236710	1239K	36.4	I	B	37	
36	1408-1413K	1412509	1413K	36.5L	D	B	64	
36	1413-1419K	1415314	1413K	36.5R	D	T	58	snoRNAs_at_1555K
36	1608-1615K	1611446	1608K	36.6	R	T	66	5'_to_tRNA_at_1608K
36	2075-2081K	2077650	2081K	36.7	I	B	50	
36	2462-2469K	2466929	2469K	36.8	R	B	102	3'_to_tRNA_cluster_at_2469K
36	2672-2679K	2677083 & 2678698	2679K	36.9	T	B	76	

**D**	**114**
**C**	**0**
**T**	**16**
**R**	**16**
**I**	**38**
**total**	**184**
**repeats**	**4**
