# Supplementary material for: Histone acetylations mark origins of polycistronic transcription in Leishmania major
Source: BMC Genomics. 2009 Apr 8;10:152. doi: 10.1186/1471-2164-10-152 (PMC2679053; doi:10.1186/1471-2164-10-152)

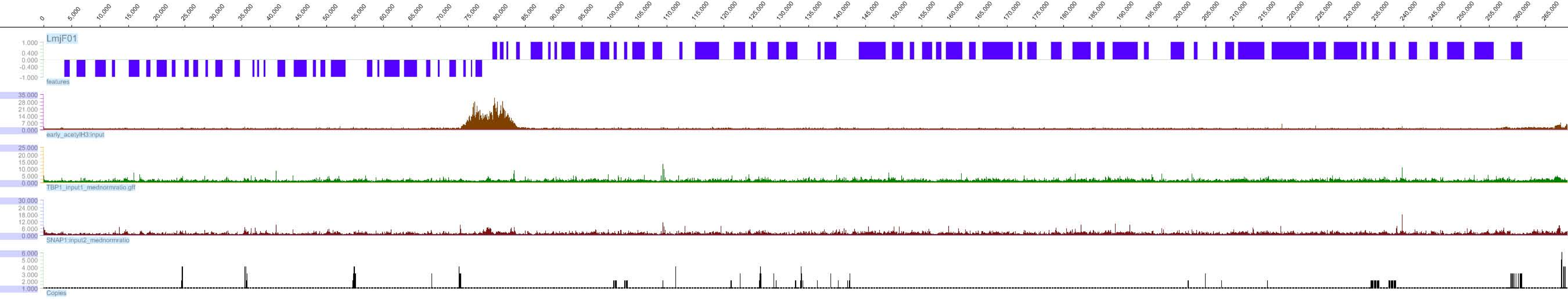
LmjF01
features
early_acetylH3:input
TBP1_input1_mednormratio.gff
SNAP1:input2_mednormratio
Copies

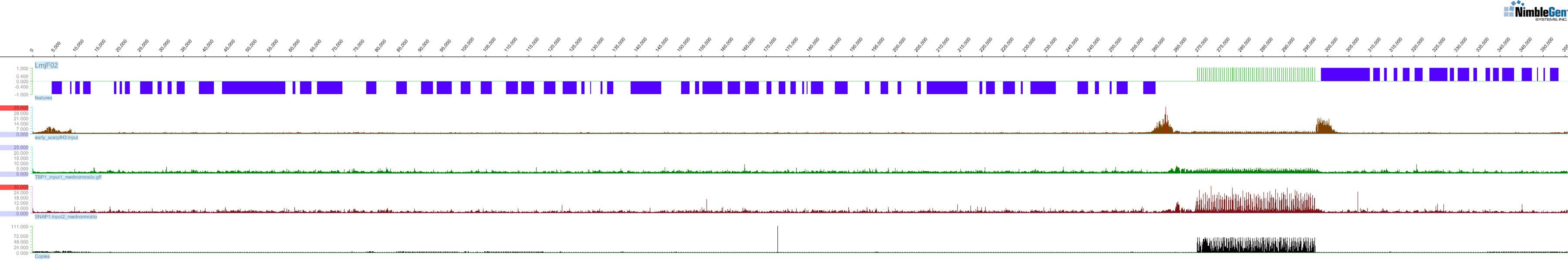
NimbleGen SYSTEMS, INC.
LmjF02
features
early_acetylH3:input
TBP1_input1_mednormratio.gff
SNAP1:input2_mednormratio
Copies

NimbleGen SYSTEMS, INC.

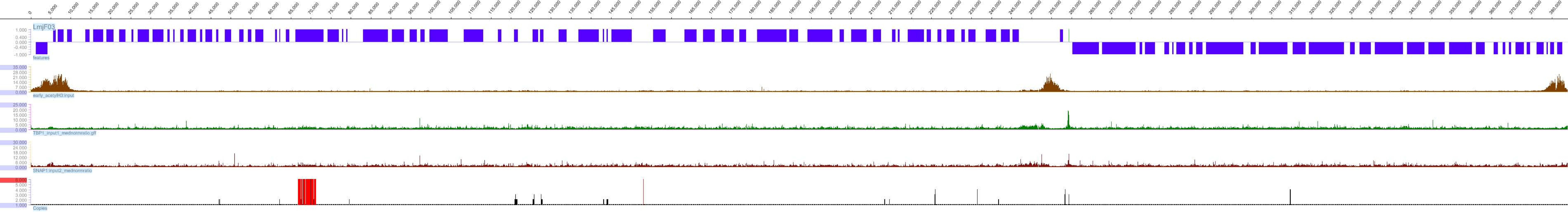

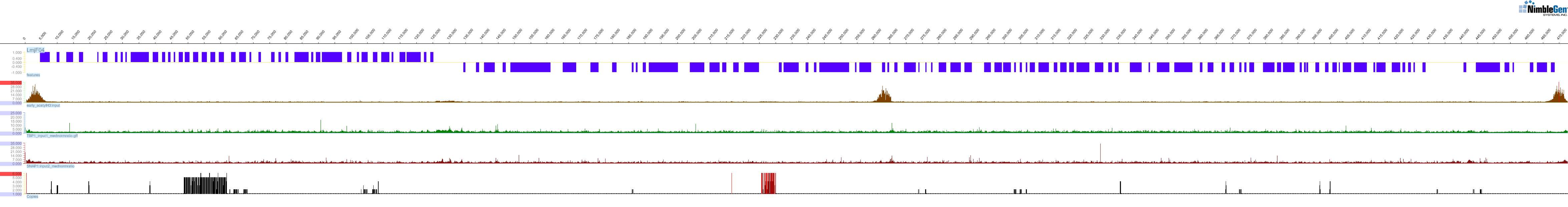

NimbleGen
LmjF04
features
early_acetylH3.input
TBP1_input1_mednormratio.gff
SNAP1.input2_mednormratio
Copies

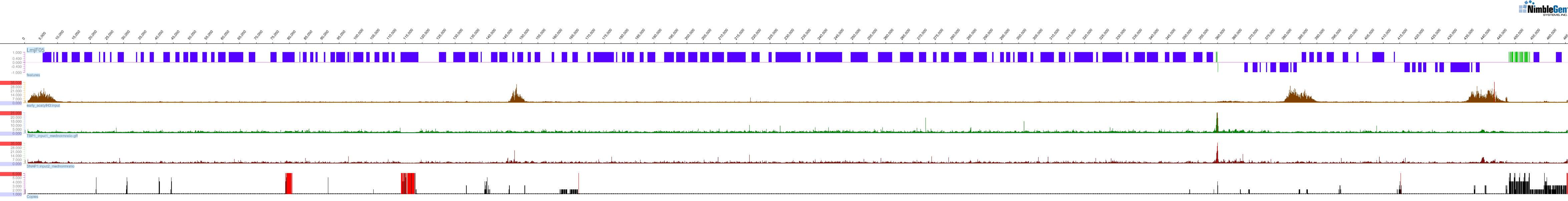
NimbleGen
LmjF05
features
early_acetylH3:input
TBP1_input1_mednormratio.gff
SNAP1:input2_mednormratio
Copies

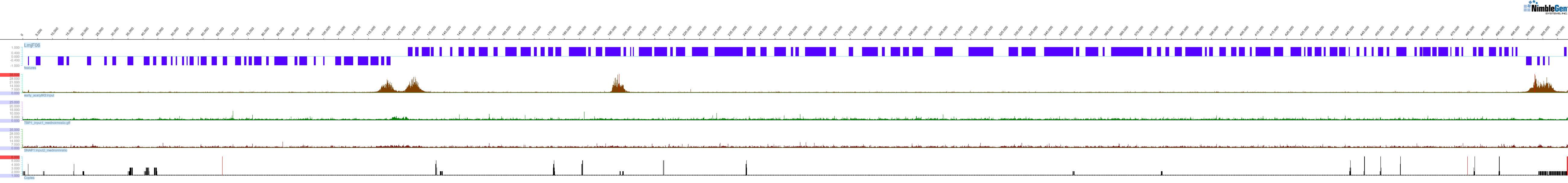

LmjF06
features
early_acetylH3:input
TBP1_input1_mednormratio.gff
SNAP1:input2_mednormratio
Copies

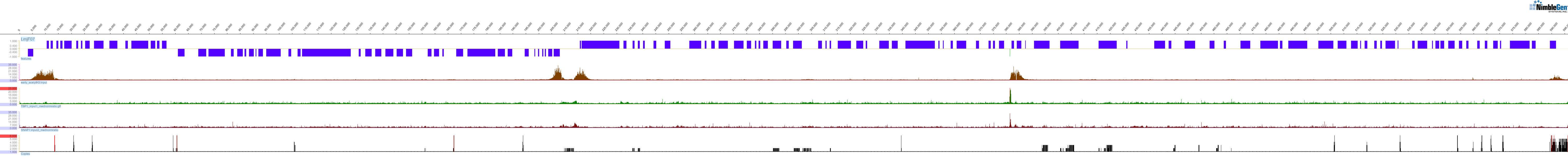
NimbleGen
LmjF07
features
early_acetylH3:input
TBP1_input1_mednormratio.gff
SNAP1:input2_mednormratio
Copies

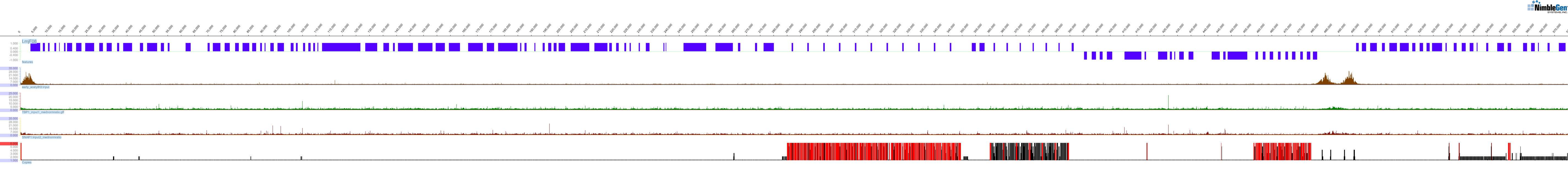
NimbleGen
LmjF08
features
early_acetylH3:input
TBP1_input1_mednormratio.gff
SNAP1:input2_mednormratio
Copies

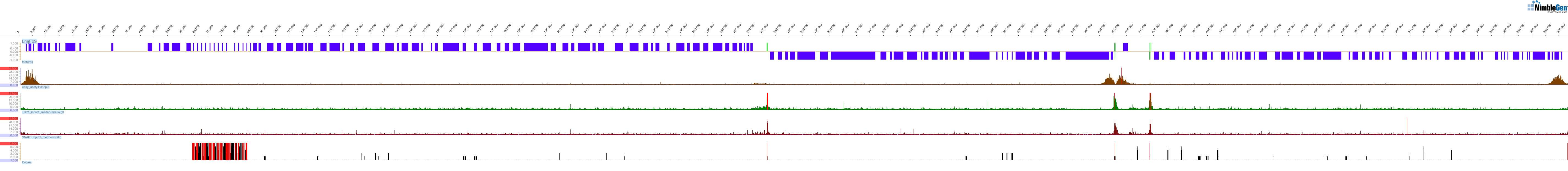

NimbleGen
LmjF09
features
early_acetylH3:input
TBP1_input1_mednormratio.gff
SNAP1:input2_mednormratio
Copies

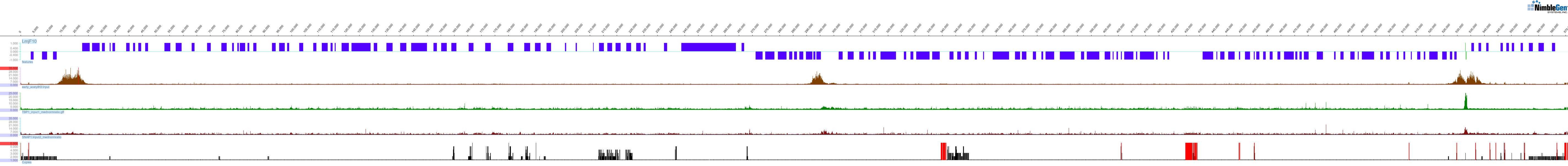
NimbleGen
LmjF10
features
early_acetylH3:input
TBP1_input1_mednormratio.gff
SNAP1:input2_mednormratio
Copies

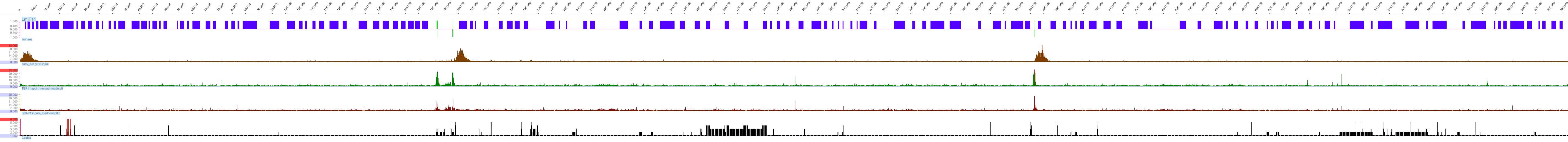
LmjF11
1.000
0.400
0.000
-0.400
-1.000
features
35.000
28.000
21.000
14.000
7.000
0.000
early_acetylH3:input
25.000
20.000
15.000
10.000
5.000
0.000
TBP1_input1_mednormratio.gff
35.000
28.000
21.000
14.000
7.000
0.000
SNAP1:input2_mednormratio
6.000
5.000
4.000
3.000
2.000
1.000
Copies

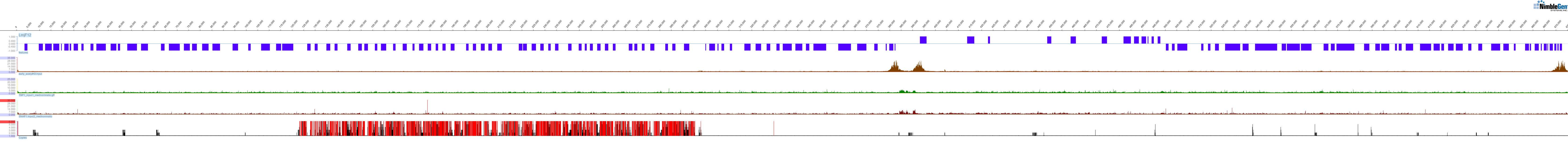

NimbleGen
LmjF12
features
early_acetylH3:input
TBP1_input1_mednormratio.gff
SNAP1:input2_mednormratio
Copies

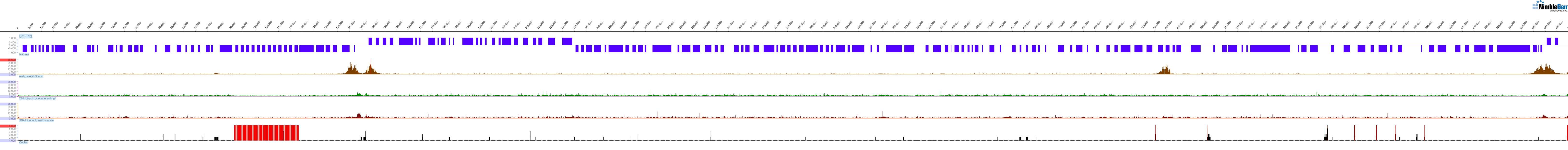
LmjF13
features
early_acetylH3:input
TBP1_input1_mednormratio.gff
SNAP1:input2_mednormratio
Copies

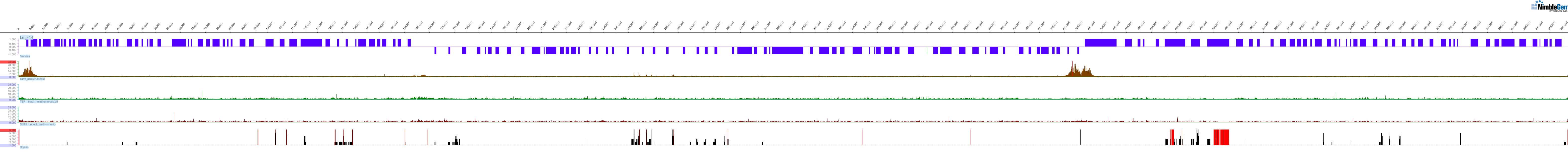
NimbleGen
LmjF14
features
early_acetylH3:input
TBP1_input1_mednormratio.gff
SNAP1:input2_mednormratio
Copies

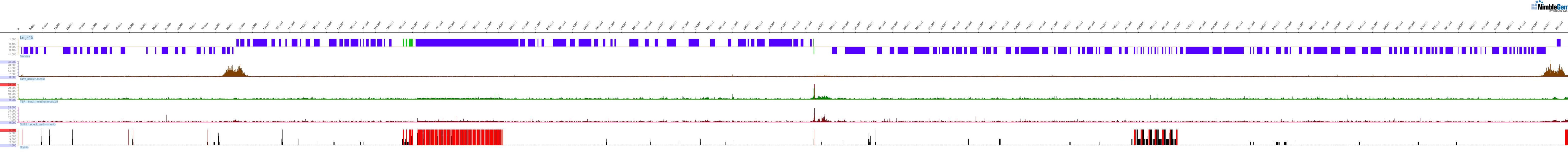
NimbleGen
LmjF15
features
early_acetylH3:input
TBP1_input1_mednormratio.gff
SNAP1:input2_mednormratio
Copies

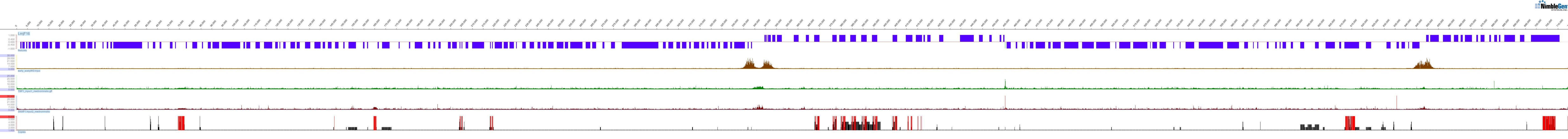
LmjF16
features
early_acetylH3:input
TBP1_input1_mednormratio.gff
SNAP1:input2_mednormratio
Copies

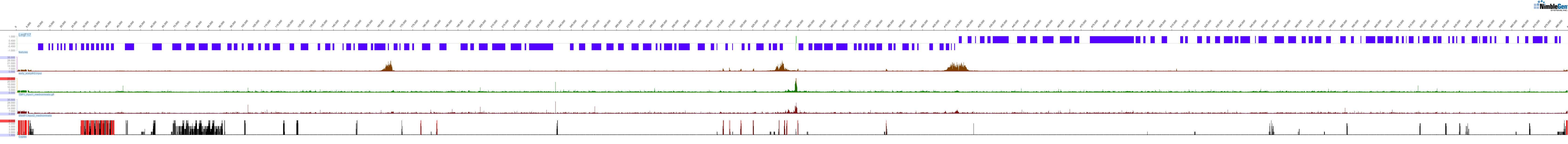

LmjF17
features
early_acetylH3:input
TBP1_input1_mednormratio.gff
SNAP1:input2_mednormratio
Copies

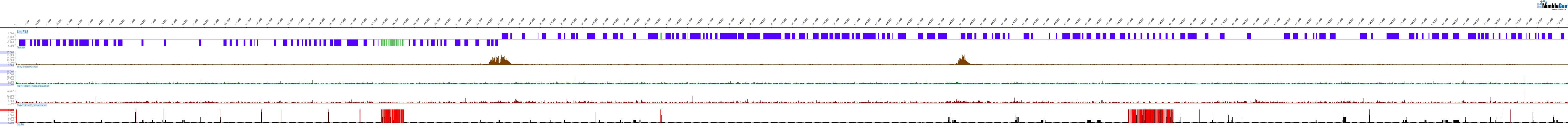

NimbleGen
LmjF18
features
early_acetylH3:input
TBP1_input1_mednormratio.gff
SNAP1:input2_mednormratio
Copies

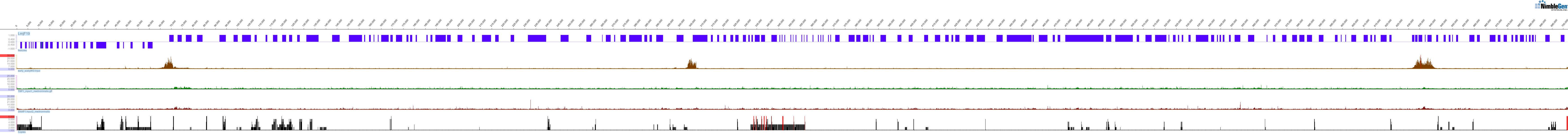
LmjF19
features
early_acetylH3:input
TBP1_input1_mednormratio.gff
SNAP1:input2_mednormratio
Copies
NimbleGen

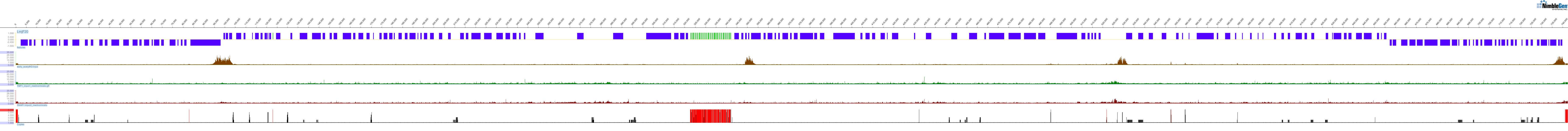

NimbleGen
LmjF20
features
early_acetylH3:input
TBP1_input1_mednormratio.gff
SNAP1:input2_mednormratio
Copies

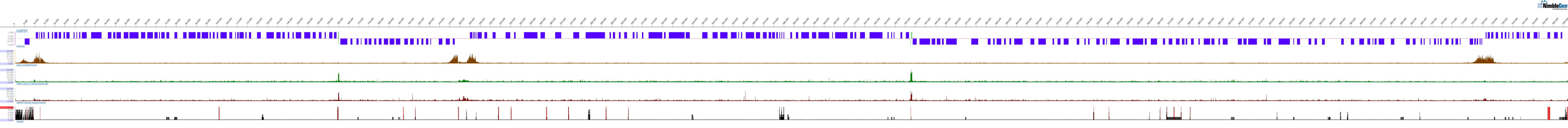

LmjF21
features
early_acetylH3:input
TBP1_input1_mednormratio.gff
SNAP1:input2_mednormratio
Copies

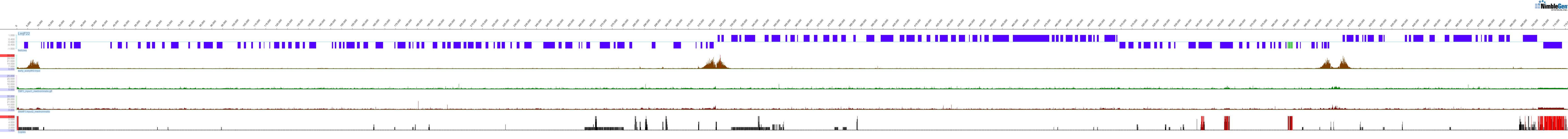

NimbleGen
LmjF22
features
early_acetylH3:input
TBP1_input1_mednormratio.gff
SNAP1:input2_mednormratio
Copies

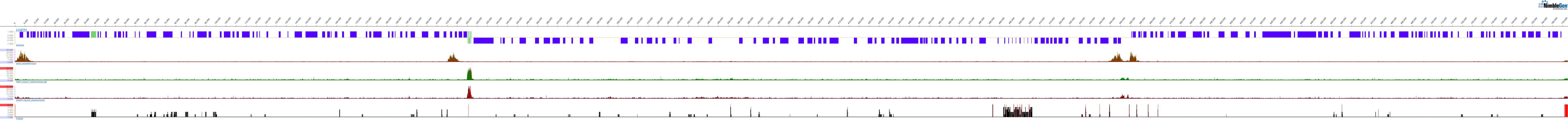

NimbleGen
LmjF23
features
early_acetylH3:input
TBP1_input1_mednormratio.gff
SNAP1:input2_mednormratio
Copies

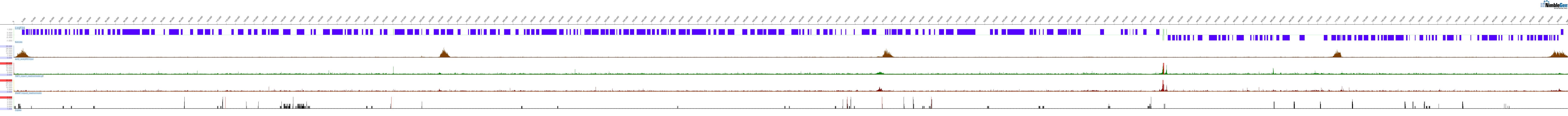

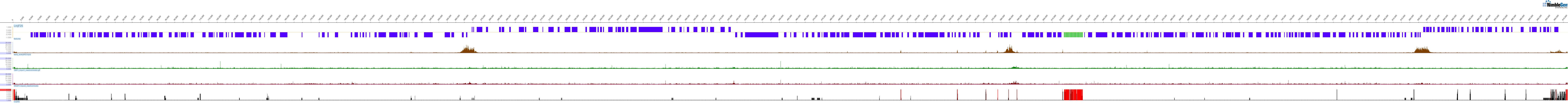
LmjF25
features
early_acetylH3:input
TBP1_input1_mednormratio.gff
SNAP1:input2_mednormratio
Copies

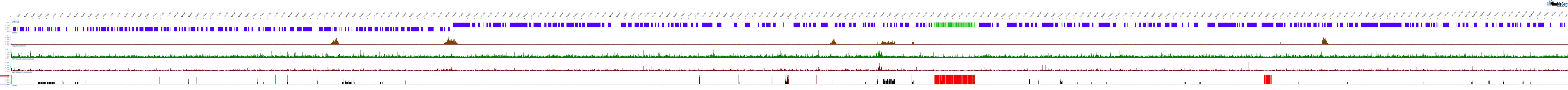

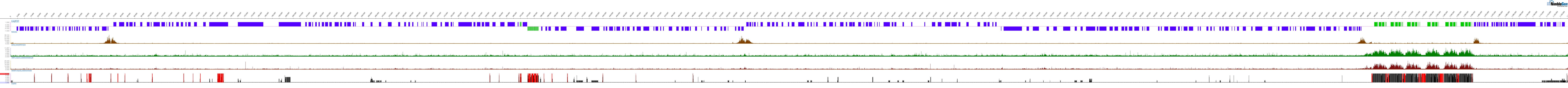

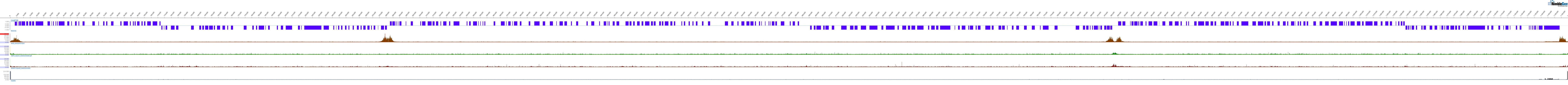

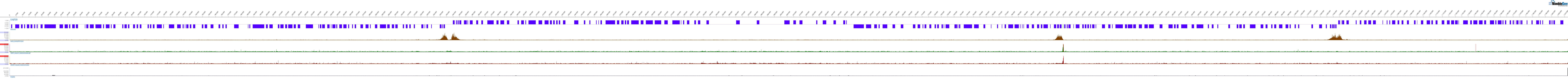

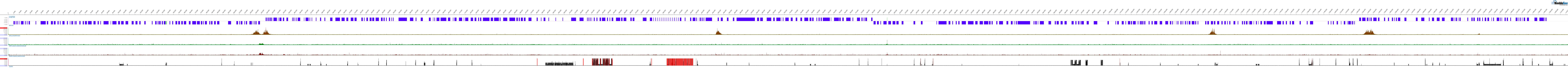

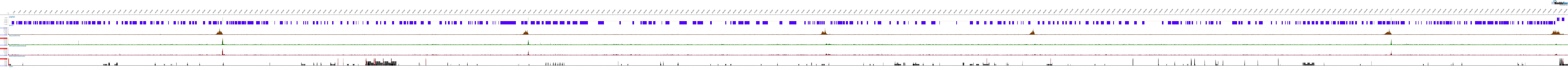

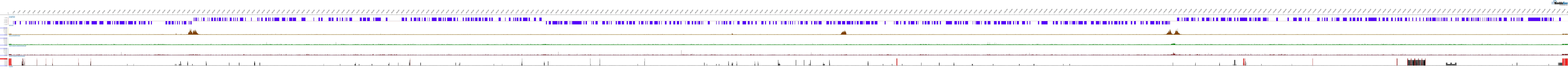

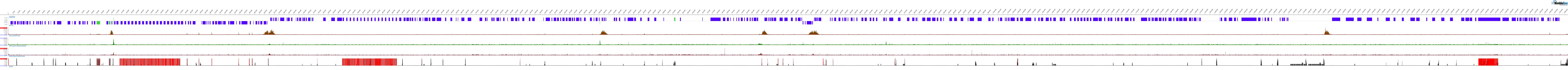

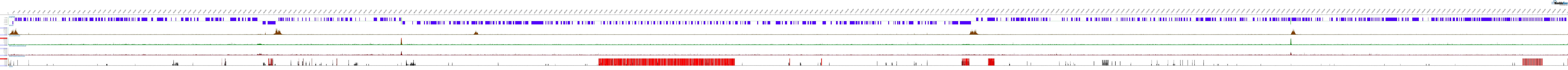
LmjF34
features
early_acetylH3:input
TBP1_input1_mednormratio.gff
SNAP1:input2_mednormratio
Copies

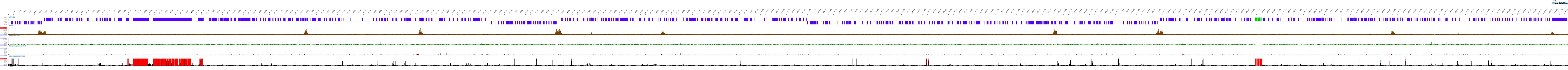

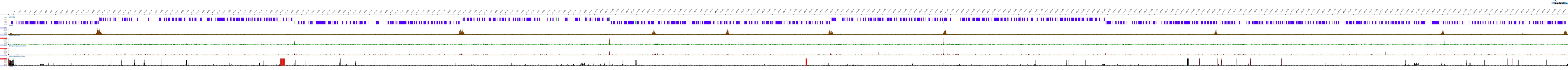

Supplement: Additional file 1 — Genome-wide distribution of acetylated histone H3, TBP, and SNAP50. The ratio of ChIP enrichment for histone H3 acetylations, TBP-binding, and SNAP50-binding are presented relative to input control for all 36 chromosomes, in similar manner to Fig 2. Also included on the bottom row of each figure is the repeat number of each oligonucleotide probe. On the top track, protein-coding genes and non-coding RNAs are shown in blue and green, respectively. A red bar at any position indicates that the value of the signal at that position is greater than the maximum value shown on the graph. [file 1471-2164-10-152-S1.pdf]
